# Neurological disorders-associated anti-glycosphingolipid IgG-antibodies display differentially restricted IgG subclass distribution

**DOI:** 10.1038/s41598-020-70063-5

**Published:** 2020-08-04

**Authors:** Ricardo D. Lardone, Fernando J. Irazoqui, Gustavo A. Nores

**Affiliations:** 1 Departamento de Química Biológica Ranwel Caputto, Facultad de Ciencias Químicas, Universidad Nacional de Córdoba, Ciudad Universitaria, X5000HUA Córdoba, Argentina; 2 Centro de Investigaciones en Química Biológica de Córdoba, CIQUIBIC, CONICET, Universidad Nacional de Córdoba, X5000HUA Córdoba, Argentina

**Keywords:** Neurological disorders, Glycoconjugates, Autoimmunity

## Abstract

Antibodies against several self-glycans on glycosphingolipids are frequently detected in different neurological disorders. Their pathogenic role is profusely documented, but the keys for their origin remain elusive. Additionally, antibodies recognizing non-self glycans appear in normal human serum during immune response to bacteria. Using HPTLC-immunostaining we aimed to characterize IgM and IgG subclass antibody responses against glycosphingolipids carrying self glycans (GM1/GM2/GM3/GD1a/GD1b/GD3/GT1b/GQ1b) and non-self glycans (Forssman/GA1/“A” blood group/Nt7) in sera from 27 randomly selected neurological disorder patients presenting IgG reactivity towards any of these antigens. Presence of IgG2 (*p* = 0.0001) and IgG1 (*p* = 0.0078) was more frequent for IgG antibodies against non-self glycans, along with less restricted antibody response (two or more simultaneous IgG subclasses). Contrariwise, IgG subclass distribution against self glycans showed clear dominance for IgG3 presence (*p* = 0.0017) and more restricted IgG-subclass distributions (i.e. a single IgG subclass, *p* = 0.0133). Interestingly, anti-self glycan IgG antibodies with simultaneous IgM presence had higher proportion of IgG2 (*p* = 0.0295). IgG subclass frequencies were skewed towards IgG1 (*p* = 0.0266) for “anti-self glycan A” subgroup (GM2/GM1/GD1b) and to IgG3 (*p* = 0.0007) for “anti-self glycan B” subgroup (GM3/GD1a/GD3/GT1b/GQ1b). Variations in players and/or antigenic presentation pathways supporting isotype (M-G) and IgG-subclass pattern differences in the humoral immune response against glycosphingolipids carrying non-self *versus* self-glycans are discussed.

## Introduction

Human sera commonly display anti-glycan antibodies (i.e. antibodies recognizing saccharide sequences on one or more types of glycoconjugates, regardless of the inducer immunogen)^1^. Certain membrane lipids (known as glycosphingolipids) exhibit oligosaccharides as their hydrophilic head groups, granting access for binding by viruses, toxins and antibodies^2^. Normal subjects routinely display naturally-occurring antibodies recognizing non-self glycosphingolipids: archetypal examples are the ABO blood group agglutinins, arisen when blood group “0” individuals develop antibodies able to agglutinate blood group “A”/“B” red blood cells^3^. These antibodies appear within the context of a normal immune response against bacteria colonizing intestinal or respiratory tract^4^. Our laboratory described a similar origin for IgM antibodies recognizing a few self glycosphingolipids such as gangliosides GM1 and GD1b^5^; however, these low affinity, cross-reactive IgM are non-pathogenic (normal anti-GM1 IgM antibodies^6^). On the contrary, autoimmune diseases frequently exhibit immune reactivity towards self-glycosphingolipids^7^. In particular, gangliosides (glycosphingolipids abundantly found in nervous system) are often targeted by antibodies present in a variety of neurological diseases^8^. Multiple triggering mechanisms for nervous system dysfunction elicited by anti-ganglioside antibodies have been well documented: formation of a membrane attack complex (MAC) at motor nerve terminals by complement activation on the nerve cell membrane, impairment of axonal membrane properties at the nodes of Ranvier causing disfunction of voltage-gated sodium channels (Nav) and conduction block; induction of apoptotic cascade activation in dorsal root ganglion cells; blockade of neurotransmitter release at motor nerve terminals by presynaptic inhibitory effect on voltage-gated Ca channel currents; complement-independent function alteration of certain receptors at lipid rafts acting as signaling platforms; and so forth^9^. Remarkably, the origin of anti-ganglioside antibodies is much less clear. Molecular mimicry between lipopolysaccharides from specific *Campylobacter jejuni* serotypes and ganglioside structures can either cause Guillain Barré syndrome (by inducing anti-GM1 and anti-GD1a antibodies) or Miller-Fisher syndrome (by inducing anti-GQ1b antibodies)^10–12^. Intriguingly, only a small fraction of individuals develops further neuropathy after infection with proper C*. jejuni* serotypes, implying additional bacterial or host constraints^13,14^. For anti-GM1 antibodies, we have proposed this additional requirement to be randomly elicited mutations affecting the binding site of normal anti-GM1 IgM antibodies (“binding site drift” hypothesis)^15^. Altogether, “molecular mimicry” and “binding site drift” hypotheses complement each other to depict how neuropathy-associated IgM and IgG anti-GM1 antibodies originate^16^.

Although recent work on IgM and IgG isotypes has extended this view to explain the origin of other anti-self glycosphingolipid antibodies associated with neurological disorders^17^, some questions persist. These types of IgG antibodies are absent in healthy humans^6,17^. Polysaccharides, some other nonprotein antigens (e.g. glycosphingolipids), and few proteins (e.g. flagellin) are regarded as T-cell independent (TI) antigens: i.e. they are able to activate B-1b and splenic marginal zone (MZ) B cells without intracellular processing and lacking assistance from CD4 + T helper (Th) cells^18^. B-1b or splenic MZ B cells exposed to cytokines such as B-cell activating factor (BAFF) and a proliferation-inducing ligand (APRIL)—generated mostly by dendritic cells—can undergo antibody class switching^19^. In contrast, most proteins are internalized by antigen-presenting cells (B-2 cells, macrophages, and dendritic cells), digested into peptide fragments and combined with MHC-class molecules to form MHC-peptide complexes that are displayed on the surface of the antigen-presenting cells to be recognized by Th-cell receptors (TCR). The specific recognition activates the B-2 cells (linked recognition), inducing antibody production and class switching. Human IgG isotype response is in turn divided into four subclasses (1 to 4) with different heavy chains influencing their own properties (e.g. Fc receptor affinity) and biological functions (e.g. complement system activation ability)^20^. Total IgG subclass levels in autoimmune disease patients do not differ substantially from those measured in healthy individuals; however, certain specific antibodies can exhibit variable subclass restrictions^21–23^. Generally speaking, IgG1 and IgG3 subclasses are mainly elicited against protein antigens, whereas certain glycan antigens preferentially induce IgG2 responses^24^. While antigen nature can have an impact on the type of IgG subclass elicited^25^, IgG subclass can also depend on the type of T helper cell (Th) response^26^. Th1 cells produce interferon-gamma (IFN-γ) and interleukin (IL) 2,Th2 cells produce IL-4 and IL-5^27^, and Th17 cells produce IL-17, IL-21, and IL-22^28^. Nevertheless, distinction between Th1, -2 and -17 cells is less pronounced in humans than in experimental mouse models^29^. Prior studies on neuropathy-associated anti-GM1, anti-GD1a and anti-GQ1b IgG antibodies indicate predominance for IgG1, IgG3 or both^30–33^; however, these studies lacked simultaneous evaluation of subclass distribution against non-self glycans for comparison purposes. In the present work, we assessed IgG-subclass humoral immune response against various self and non-self glycan-carrying glycosphingolipids in patients with diverse neurological disorders. Reactivity pattern differences were thoroughly analyzed in the context of potential origin diversity for these antibodies.

## Results

### IgG antibody subclass distribution is different between responses against self glycan- and non-self glycan-carrying glycosphingolipids

**Figure 1:**
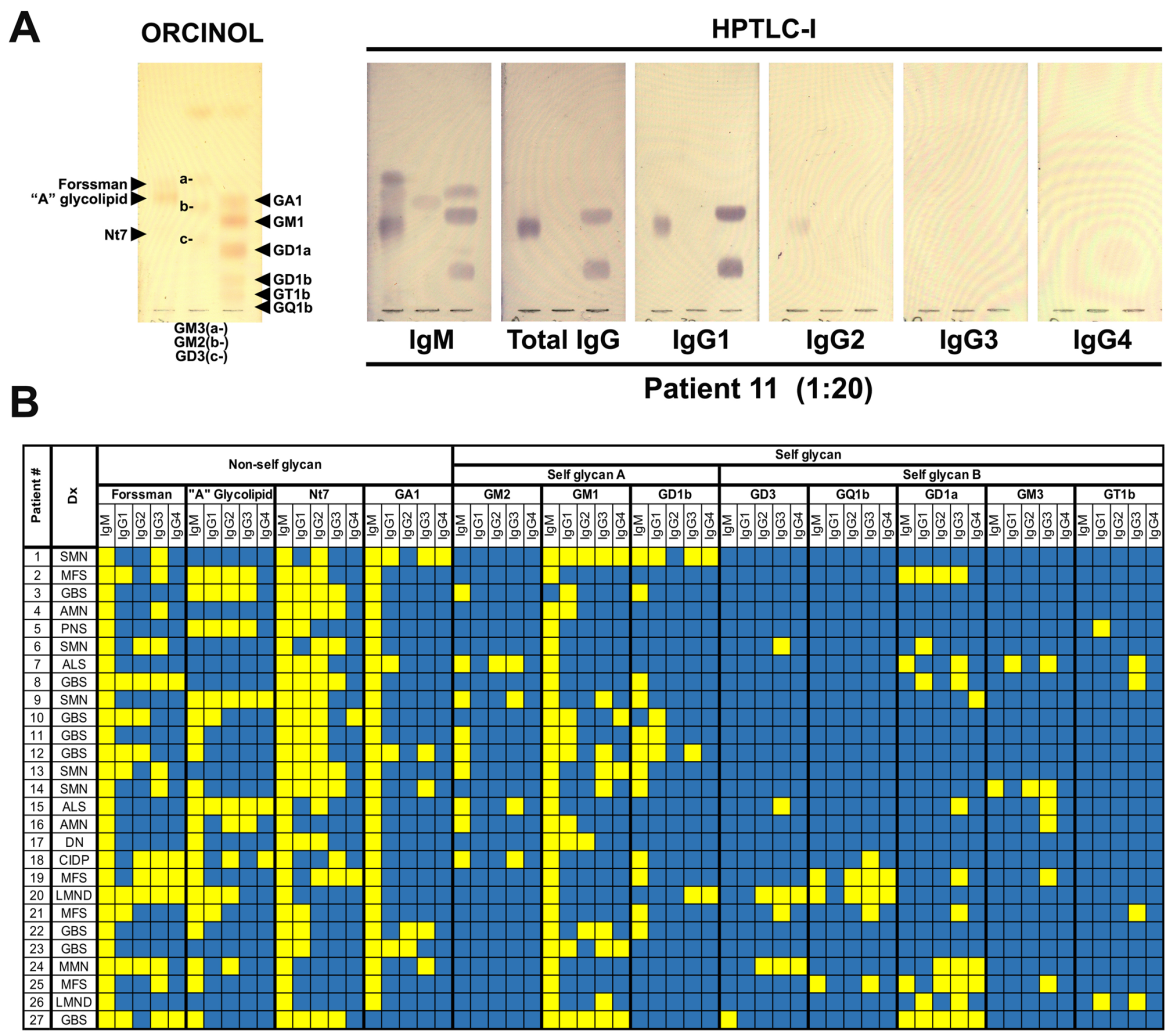
Distribution of anti-non-self glycan and anti-self glycan IgM and IgG subclass antibodies in neurological disorder patients. **(A)** A representative HPTLC-I result, corresponding to patient # 11. After serum incubation, proper specific secondary antibody (see M&M) for binding detection of each isotype (IgM or IgG) or each IgG subclass (IgG1, IgG2, IgG3 or IgG4) was added. On left plate the different glycosphingolipids were visualized with orcinol reagent. **(B)** Summary of anti-non-self glycan and anti-self glycan IgM and IgG subclasses found in 27 randomly chosen, anti-self glycan IgG Ab-positive, neurological disorder patients. Presence (yellow squares) or absence (blue squares) of IgM and IgG antibody subclasses reactive for each glycosphingolipid (by HPTLC-I) is shown. Patient number (Patient #) and neurological disorder diagnosis (Dx) are detailed in far-left columns. *ALS* amyotrophic lateral sclerosis, *AMN* asymmetric motor neuropathy, *CIDP* chronic inflammatory demyelinating polyneuropathy, *DN* diabetic neuropathy, *GBS* Guillain-Barré syndrome, *LMND* lower motor neuron disease, *MFS* Miller Fisher syndrome, *MMN* multifocal motor neuropathy, *PNS* paraneoplastic syndrome, *SMN* sensory-motor neuropathy.

We comparatively analyzed the percentage distribution for anti-non-self glycan and anti-self glycan IgG-subclass antibodies in 27 randomly selected patient serum samples (positive for diverse anti-self glycan IgG antibodies) by HPTLC-I. Figure 1A shows patient # 11 serum analysis as an example: anti-non-self glycan reactivity comprised anti-Forssman antibodies (IgM), anti-“A” glycosphingolipid antibodies (IgM), anti-Nt7 antibodies (IgM, IgG1 and IgG2), and anti-GA1 antibodies (IgM). Anti-self glycan reactivity was formed by anti-GM2 (IgM), anti-GM1 (IgM and IgG1) and anti-GD1b (IgM and IgG1) antibodies. These analyses were repeated for all patient serum samples evaluated (see Supplementary Figure S1). Figure 1B summarizes the different distributions of anti-self and anti-non self-glycan IgG-subclass antibodies for each neurological disorder patient. When necessary, inhibition experiments using soluble self glycan-carrying glycosphingolipids were performed to confirm or discard differences in IgM and IgG fine specificities (results not shown). Initial evaluation of HPTLC-I results indicated the observed patterns of anti-self glycan IgG antibodies correlated with the specific diseases for some of the neurological disorders most represented in our samples: IgG anti-GM1 in Guillain Barré syndrome (7 of 8; 88%) and IgG anti-GQ1b in Miller Fisher syndrome (3 of 4; 75%)^8^.

**Figure 2:**
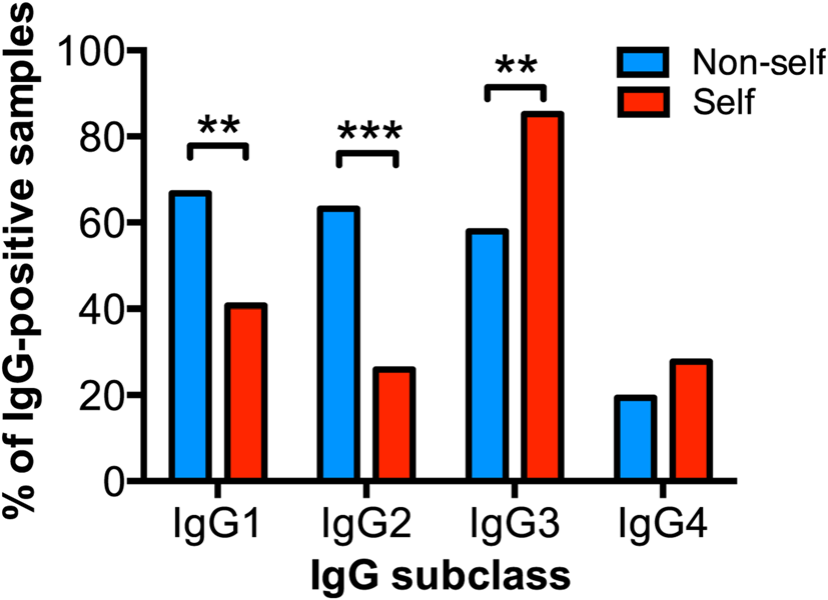
Anti-self glycan antibodies have a different IgG subclass distribution compared to those against non-self glycans. Percentage of samples having antibody reactivity against antigens grouped in non-self glycan (blue bars) and self glycan (red bars) are shown for each IgG subclass. The different anti-non-self glycan and anti-self glycan IgG subclass antibody reactivities were determined using HPTLC-I (**: *p* < 0.01; ***:*p* < 0.001; Fisher's exact test).

For IgG subclass distribution analyses of each given antigen group, we plotted the percentage of serum samples positive for each IgG subclass (considering the number of samples positive for any IgG subclass towards that antigen as the 100%; see Materials and Methods). Anti-non self glycan IgG antibodies had a significantly higher frequency of IgG1 (*p* = 0.0078) and IgG2 (*p* = 0.0001). In contrast, sera positive for anti-self glycan IgG antibodies showed a more frequent presence of IgG3 (*p* = 0.0017; Fig. 2).

**Figure 3:**
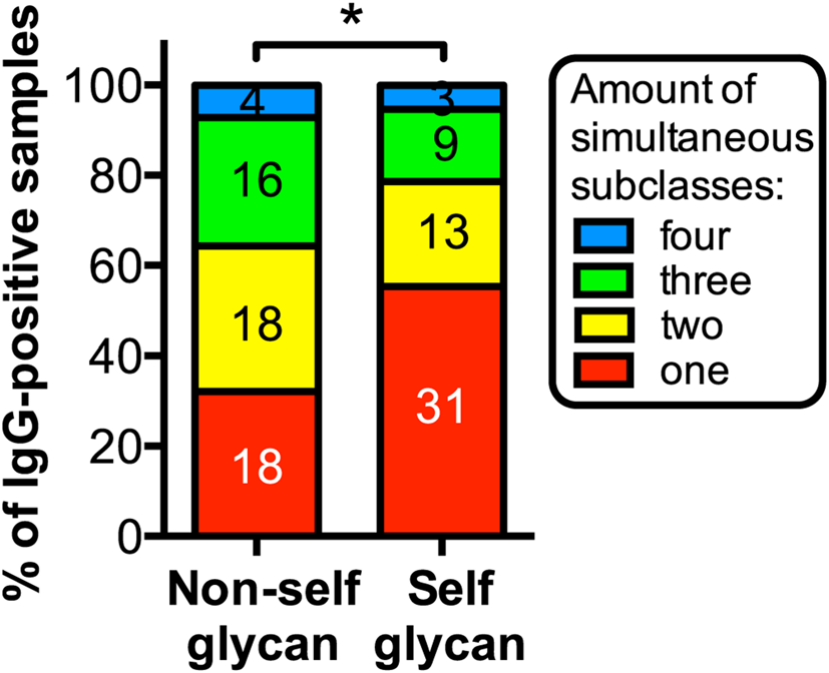
The reactivity against self glycans is associated to a more restricted IgG subclass antibody response. Stacked bars show proportion of IgG antibody populations composed by single (“one”) versus multiple different (“two”, “three” or “four”) IgG subclasses in anti-non-self glycan and anti-self glycan IgG antibody responses. The presence of single IgG subclass populations is considered indicative of a more restricted response (*: *p* < 0.05, Fisher’s exact test).

Examined in close detail across the different serum samples, some IgG antibody responses comprised two or more different IgG subclasses, whereas others were formed by a single IgG subclass. The reactivity against self glycans was significantly associated to the presence of a single IgG subclass (*p* = 0.0133), thus representing a more restricted antibody response compared to that towards non-self glycans (Fig. 3).

### Changes in IgG antibody subclass distribution within responses against different self glycan-carrying glycosphingolipids

**Figure 4:**
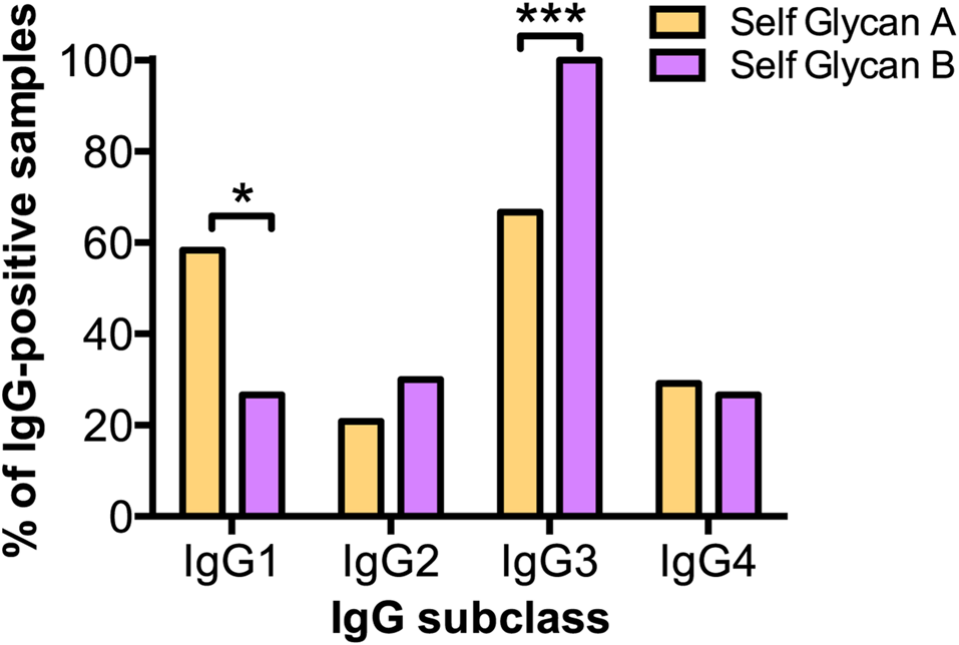
The IgG subclass frequencies are skewed towards different IgG subclasses between anti-self glycan A and anti-self glycan B responses. IgG subclass frequency distributions for antigens grouped in self glycan A (peach bars) and self glycan B (purple bars) are shown. Antibody reactivities from each subclass were determined using HPTLC-I. (**: p < 0.01; ***: p < 0.001; Fisher's exact test).

IgM populations recognizing certain self glycan-carrying glycosphingolipids (GM2/GM1/GD1b) have been reported in normal human sera^6^, leaving room for potential antibody response differences within the various self glycan glycosphingolipids tested. Based on this, reactivities against anti-self glycan glycosphingolipids were subgrouped in “anti-self glycan A” (GM2/GM1/GD1b) and “anti-self glycan B” (GD3/GQ1b/GD1a/GM3/GT1b, see “Methods”). The proportion of IgG1 subclass antibody response was significantly higher for anti-self glycan A than for anti-self glycan B subgroup (*p* = 0.0266). On the contrary, anti-self glycan B subgroup reached a significantly higher IgG3 proportion compared to anti-self glycan A subgroup (*p* = 0.0007; Fig. 4), supporting the notion for a distinct type of IgG response within the antibodies against self glycans.

### The presence of anti-self glycan IgM antibody counterpart is associated with differences in anti-self glycan IgG antibody subclasses

**Figure 5:**
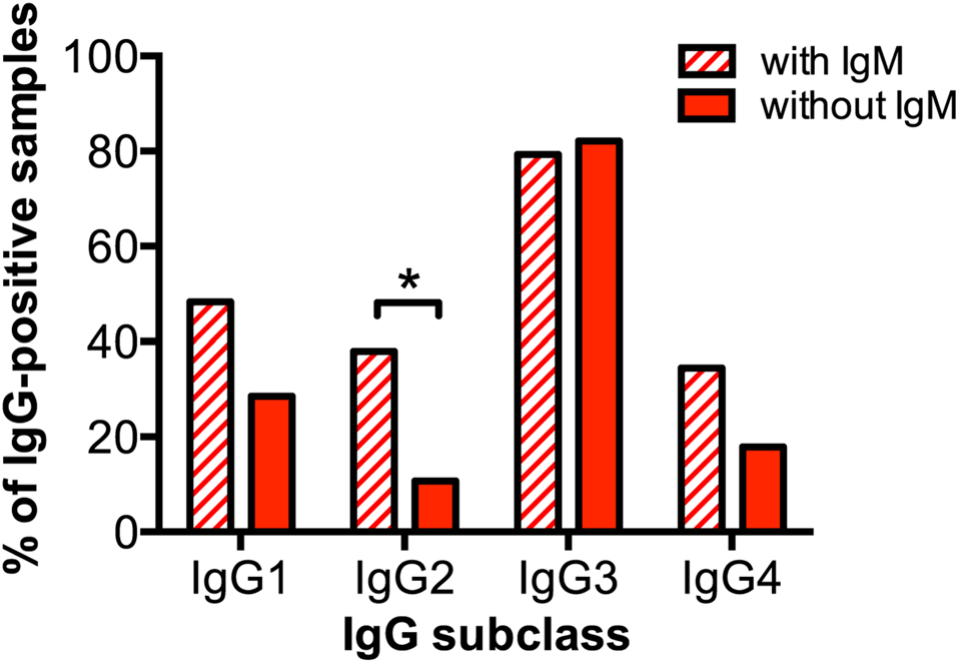
IgM counterpart presence for anti-self glycan IgG antibody responses is associated with an increased frequency of IgG2 subclass. Antibody reactivities of anti-self glycan antibodies detected along with IgM (striped bars) versus anti-self glycan antibodies without IgM counterpart (red-filled bars) were determined for each subclass using HPTLC-I. *: *p* < 0.05 (Fisher’s exact test).

Irrespective of their specificity, some populations of anti-self glycan IgG antibodies were present along with their IgM counterpart, while others had their IgM counterpart absent (Fig. 1B). We therefore grouped anti-self glycan IgG antibody subclass results in different antibody subpopulations based on the simultaneous presence or absence of IgM counterpart. Anti-self glycan IgG responses present along with their IgM counterpart displayed a higher proportion of IgG2 compared to those IgG responses without IgM counterpart (*p* = 0.0295; Fig. 5).

## Discussion

Even though invertebrates can distinguish self- from non-self components, it is not until the level of terrestrial vertebrates—amphibians, reptiles, birds, and mammals—that a complete immune system with thymus, spleen, bone marrow, and lymph nodes is present and IgM and IgG antibodies are made^34^. Besides their crucial role in host defense and homeostasis, improper control of antibody production can generate antibodies towards certain self-antigens (such as glycan antigens) and cause autoimmune diseases^8^. The structural similarities between non-self and self glycans could indicate connections between both responses^16^. In the present work, we characterized the antibody immune response (IgM, total IgG and IgG subclasses) against diverse non-self and self glycosphingolipids in a cohort of patients with neurological disorders. Parallel evaluation of the humoral immune response against glycosphingolipids carrying either non-self glycans or self glycans allowed comparisons between both types of responses, where anti-non-self glycan IgM antibodies are considered anti-bacterial antibodies^4,5,35^.

As already observed, anti-non-self glycan IgG antibodies always had their IgM counterpart^17^, and presented a less restricted IgG subclass response (i.e. two or more different IgG subclasses). Displaying IgG2 and IgG1 as prevailing IgG subclasses, they resembled antibody responses against bacterial glycans^36,37^, with IgG2 hinting at a relevant contribution of a TI immune response to these antibody populations^38^. On the contrary, IgG antibodies against self glycans frequently exhibited a more restricted IgG-subclass distribution (i.e. a single IgG subclass) and an increased proportion of IgG3 subclass (consistent with a T-cell dependent (TD) immune response^39^.

With different biological features, three subsets of naïve B cells craft normal antibody repertoire populations after differentiation into plasma cells: B-1 B cells (typically subdivided into B-1a and B-1b B cells), follicular (FO) B cells, and MZ B cells^40^. B-1 B cells (residing mainly in the peritoneal and pleural cavities) produce IgM antibodies directed against T-independent (TI) antigens like carbohydrate or phospholipid antigens^41^. FO and MZ B cells (often referred to as B-2 cells) are present in secondary lymphoid organs. Although both B-2 populations can experience Ig class switching and differentiate into memory cells^40,42^, FO B cells are primarily responsible for generating long-lasting, high affinity IgG antibodies against T-dependent antigens^40^, while MZ B cells can also recognize TI carbohydrate and phospholipid antigens (like B-1 cells do)^42^. TI type II immune responses generate memory B cells^43^. IgM-expressing memory B cells with IgM^+^IgD^+^CD27^+^ phenotype can undergo secondary germinal center reactions upon reactivation with glycan antigens, differentiate to plasma cells and switch their isotype to IgG2^44,45^. We observed significantly more IgG2 contribution when IgM was present in anti-self glycan responses. IgG2 has lower ability to activate complement, to induce antibody-dependent cell-mediated cytotoxicity and to trigger antibody-dependent cellular phagocytosis than IgG1 and IgG3^20^: competition of IgG2 with other IgG subclasses for antigen binding in a context of IgG2 anti-self glycan relative enrichment could lead to lessen pathogenic events, and to an overall reduced severity of the neurological disorder.

Glycan-antibody interactions occur via a number of attractive forces of hydrophilic and hydrophobic nature^46^: the acquisition, modification or loss of interaction points allows emergence of clones with specificity changes. Successive B-cell activation events introducing single nucleotide changes at high speed (~ 1 mutation every 10^3^ base pairs/generation) can provide fast BCR diversification^47^ and some variants with potential affinity maturation through somatic hypermutation^48^. In the context of a process we have coined as “binding site drift” hypothesis^16^, new specificities could now lead these anti-non self glycan antibodies to cross-react with sequences similar to those from self glycan. Bacterial-driven B-cell activations can expand these anti-self glycan IgG B-cell clones. Upon reaching a certain antibody affinity threshold value, these B-cell clones can now be activated by self glycan oligosaccharide structures, either on self molecules or on foreign components from certain microorganisms (e.g. *Campylobacter jejuni* LPS O-antigen with oligosaccharide motifs similar to GM1, GD1a, GD3, GT1a, GQ1b; *Haemophilus influenzae* mimicking GM1 and GT1a; *Cytomegalovirus* resembling GM2^8^). Even though glycans are considered TI antigens, our findings regarding IgG subclasses suggest anti-self glycan responses can develop by two mechanisms: one TI (for anti-self glycan with IgM counterpart) and the other TD (for both types of anti-self glycan). T-cell involvement is indeed a likely event: MZ B cells can present lipoglycans and glycosphingolipids to CD1-restricted α/β and γ/δ T-cells. Activation via CD40L–CD40 and T cell multiplication provide the context for proliferation and antibody responses with eventual class switch^49^. That would not necessarily be detrimental for IgM: IgG antibody responses from MZ B-cells tend to maintain IgM production, even for long-term memory B-cell populations^50^.

**Figure 6:**
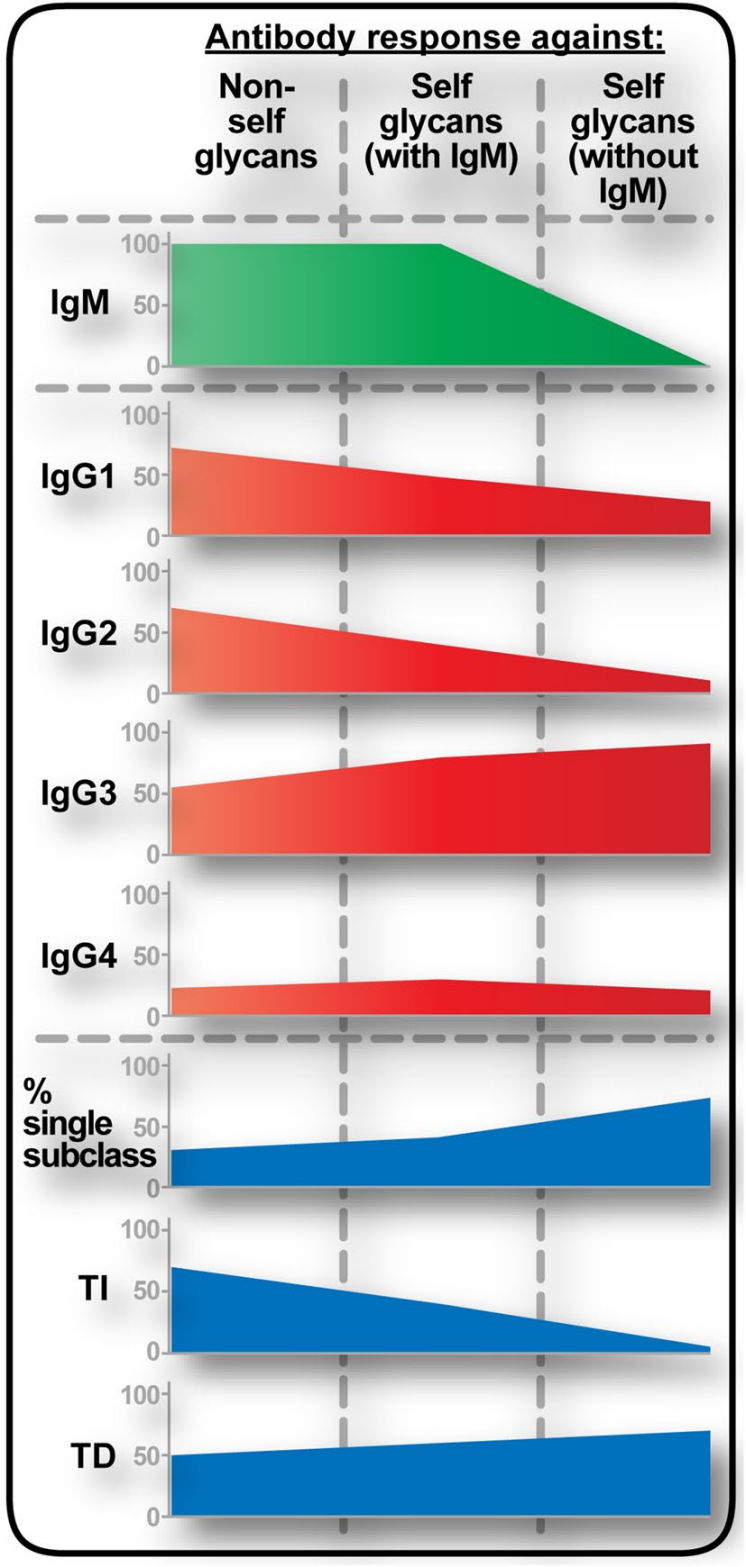
IgG antibody subclass responses are qualitatively different between self glycans and non-self glycans. Trends are displayed proportionally to percentage distributions for each isotype and subclass. *TI* T-independent antibody response, *TD* T-dependent antibody response.

How do anti-self glycan IgG antibodies occur if they have no IgM counterpart in normal human serum? One possibility would be a response against self glycan initiated by anti-self glycan (autoreactive) B-cells with surface IgM, leaving only anti-self glycan memory B-cells carrying surface IgG remaining after this first response. We hypothesize anti-self glycan IgM^+^ B-cell clones populations that experienced a “binding site drift” process and are activated by self glycan would have the chance to expand. Some selected B-cell clones could then change their isotype to some of the different IgG subclasses, overall resulting in two or more IgG subclasses elicited in addition to IgM. On the contrary, clones originated from a “binding site drift” event at the IgG level are already committed to an IgG subclass. Therefore, expansion of one of those clones would generate only anti-self glycan antibodies from that specific IgG subclass, with neither anti-self glycan IgM antibodies nor the remaining IgG subclasses. This could account for the less restricted IgG subclass distribution observed for anti-non-self glycan IgG antibodies, compared to anti-self glycan IgG antibodies. Implying a potential association between IgM presence, a broader IgG subclass response and a substantial TI component, these and the aforementioned findings are represented in Fig. 6.

Within anti-self glycan IgG antibody reactivity (showing increased IgG3 subclass proportion, i.e. TD response), antibodies recognizing “self glycan B” subgroup had significantly higher proportion of IgG3 than those against “self glycan A” subgroup, suggesting origin differences. It is important to note that the “self glycan B” subgroup (GM3/GD3/GD1a/GT1b/GQ1b) comprises glycosphingolipids carrying a N-Acetyl Neuraminic Acid (NeuNAc) bound to a terminal galactose (NeuNAcα2,3Gal). This is a very important factor regarding anti-self glycan antibody specificity: for example, antibodies towards GM_1_ (part of “self glycan A” subgroup) require a free terminal galactose and (in some patients) an internal NeuNAc for binding, rendering them unable to cross-react with self glycans carrying terminal NeuNAc (“self glycan B”). The inbalance in IgG1-/IgG3-subclass proportion could have consequences in pathology development, since IgG3-subclass has a better complement activator ability than IgG1^25^ albeit its three-times shorter plasma half-life ^20^. Considering antibodies against terminal NeuNAcα2,3Gal epitope have been preferentially associated with axonal damage^51^, one can speculate that the higher biological activity of IgG3 can have an influence on the greater severity and slower recovery observed in the axonal forms (compared to demyelinating variants) of Guillain-Barré syndrome^52^.

Anti-self glycan antibodies are an important component in the multifactorial process defining clinical evolution for certain neurological disorders. Evidence from GBS patients with anti-GM1 IgG antibodies indicates they do not present too many different populations simultaneously, although different patients exhibit different anti-GM1 antibody fine specificities^53^. We could consider they together represent different possibilities of antibody specificity changes and expand this perspective to other anti-self glycan IgG-mediated neurological disorders, assuming that emergence of different anti-self glycan IgG antibody populations results from a random process. As we discussed earlier, in the context of the “binding site drift” hypothesis we could potentially foresee different types of antibody responses for the same self glycan target. One response, originated from an anti-self glycan IgM^+^ B-cell population that drifted and is now activated by self glycan can lead to some IgM clones changing their isotype to produce various different IgG subclasses in combinations of TI responses (mainly IgG2) and TD responses (mainly IgG1 and IgG3). In contrast, a response generated from anti-self glycan IgG^+^ B-cell clones (already committed to a determined IgG subclass) that drifted at the IgG level (upon expansion of one of those clones in a TD response) would make only that specific IgG subclass. Based on biological activity disparities exhibited by each IgG subclass, the differences in IgG subclass responses generated could have clinical implications concerning disease severity, response to therapies and/or recovery time, as it has been observed for the slower recovery in GBS associated with anti-GM1 IgG1 antibodies^32^. Further research is warranted exploring the influence that different types of IgG subclass responses against self glycan-carrying glycosphingolipids could have in clinical evolution of other autoimmune neurological disorders.

## Methods

### Human sera

Disease serum samples were collected before any immune treatment from patients attending Neurology services from Hospital “Ramos Mejía” and Hospital Nacional de Clínicas “José de San Martín” (Buenos Aires, Argentina) with early symptoms of neurological disease. After clot separation, sera were submitted to our laboratory for routine determination of anti-glycosphingolipid antibodies (GM1, GM2, GM3, GD1a, GD1b, GD3, GT1b, GQ1b). From samples resulting positive for IgG antibodies against any of these self glycan-carrying glycosphingolipids, we randomly selected 27 patients for further analysis (amyotrophic lateral sclerosis, *n* = 2; asymmetric motor neuropathy, *n* = 2, chronic inflammatory demyelinating polyneuropathy, *n* = 1, diabetic neuropathy, *n* = 1; Guillain-Barré syndrome, *n* = 8; lower motor neuron disease, *n* = 2; Miller Fisher syndrome, *n* = 4; multifocal motor neuropathy, *n* = 1; paraneoplastic syndrome, *n* = 1; sensory-motor neuropathy, *n* = 5). With different extents, all these neurological disorders have been reported to present autoimmune components^8,54,55^. All procedures were performed in accordance with Ethical Guidelines on Research Involving Human Subjects^56^ and with ethical standards as laid down in the 1964 Declaration of Helsinki and its later amendments, with prior approval by the Ethics Committee of CIQUIBIC-CONICET. Informed consent was obtained from the patients.

### Glycosphingolipids

Glycosphingolipids were obtained from the following biological materials: Sandhoff disease human brain for GM2; dog erythrocytes for GM3; chick brain for GD3; human brain for GM1, GD1a, GD1b, GT1b, and GQ1b; sheep erythrocytes for Forssman glycosphingolipid (Forssman); human blood group "A” meconium for blood group "A" glycosphingolipid; *Calliphora vicina* pupae for Nt7 glycosphingolipid^57^. Folch upper phase from partitioned lipid extract^58^ was sequentially purified using DEAE -chromatography^59^ and HPLC on Iatrobeads silica-gel column^60^. Acid hydrolysis of cow brain gangliosides was used to prepare asialo-GM1 (GA1)^61^. Lipid purity was checked by HPTLC with orcinol reagent.

### High performance thin layer chromatography (HPTLC)-immunostaining

HPTLC with subsequent immunodetection (HPTLC-I) represents the “golden standard” to detect anti-glycosphingolipid antibodies and confirm autoreactivity results^62,63^. Using a tank designed to obtain highly reproducible chromatograms^64^, glycosphingolipids (0.3 nmoles each) were resolved on HPTLC plates in the running solvent chloroform–methanol-aqueous 0.2% CaCl_2_ (45:45:10). Plates were air-dryed and coated by dipping for 2 min in a 0.5% solution of polyisobutylmethacrylate (Plexigum P 28, Röhm and Haas, Darmstadt, Germany) in n-hexane-chloroform (9:1). After blocking with BSA-PBSt (1% bovine serum albumin in phosphate buffered saline containing 0.05% Tween 20) for 1 h, plates were incubated overnight with BSA-PBSt diluted serum (1/20) and washed thoroughly with PBSt. Binding was detected following 2 h incubation with BSA-PBSt diluted (1/1,000) peroxidase-conjugated anti-human IgM (μ chain) or IgG (γ chain) goat antibodies (Sigma, USA). For IgG subclass determination, BSA-PBSt diluted biotin-conjugated antibodies against human γ1 (IgG1), γ2 (IgG2), γ3 (IgG3) o γ4 (IgG4) chains (Sigma, USA) were incubated for 2 h, followed by 1 h incubation with PBSt-diluted peroxidase-conjugated streptavidin (Sigma, USA). All the incubation steps were performed at 4 °C. After washing, color development was achieved in a substrate solution containing 2.8 mM 4-chloro-1-naphtol and 0.01% H_2_O_2_ in methanol-20 mM Tris–HCl buffer, pH 7.4 (1:29). Plates were finally washed with PBSt after 20 min to stop the reaction. Digital images of immunostaining results were quantified using ImageJ software (1.52 version) and converted to categorical data upon setting a minimal peak area threshold of 500.

### Statistical analyses

Antibody reactivity reported as categorical data was combined into groups for statistical purposes. Immunostaining against non-self glycans (GA1, Forssman, Nt7 and blood group "A" glycosphingolipid for “0” and “B” blood group individuals) was grouped as “non-self glycan” reactivity. IgM populations against GM1, GD1b and GM2 are present in normal human sera^6^; hence, IgG reactivity against GM1, GD1b and GM2 was at times considered a subgroup (“self glycan A”). Finally, IgG response against GM3, GD3, GD1a, GT1b and GQ1b was counted as another subgroup of self glycans (“self glycan B”). For each given antigen group, IgG subclass distribution analyses were done by comparing the number of samples positive for each IgG subclass to the number of samples positive for total IgG antibodies towards that antigen (considered as the total sample number). Data were examined by Fisher’s exact test with Prism 6 (GraphPad software, La Jolla, CA). Differences with *P* value < 0.05 were considered significant. Degrees of statistical significance are presented as follows: *, *p* < 0.05; **, *p* < 0.01; ***, *p* < 0.001, or ****, *p* < 0.0001.

## Supplementary information

Supplementary information.

## Abbreviations

PBSt: Phosphate buffered saline containing 0.05% Tween 20
BSA-PBSt: 1% Bovine serum albumin in PBSt
Forssman: GalNAcα1-3GalNAcβ1-3Galα1-4Galβ1-4Glcβ1-Cer
Nt7: GlcNAcβ1-3Galβ1-3GalNAcα1-4GalNAcβ1-4GlcNAcβ1-3Manβ1-4Glcβ1-Cer
“A” blood group glycosphingolipid: GalNAcα1-(Fucα1,2)3Galβ1-4GlcNAcβ1-3Galβ1-4Glcβ1-Cer
GA1: Galβ1-3GalNAcβ1-4Galβ1-4Glcβ1-Cer
GM3: NeuNAcα2-3Galβ1-4Glcβ1-Cer
GD3: NeuNAcα2-8NeuNAcα2-3Galβ1-4Glcβ1-Cer
GM2: GalNAcβ1-(NeuNAcα2,3)4Galβ1-4Glcβ1-Cer
GM1: Galβ1-3GalNAcβ1-(NeuNAcα2,3)4Galβ1-4Glcβ1-Cer
GD1a: NeuNAcα2,3Galβ1-3GalNAcβ1-(NeuNAcα2,3)4Galβ1-4Glcβ1-Cer
GD1b: Galβ1-3GalNAcβ1-(NeuNAcα2,8NeuNAcα2,3)4Galβ1-4Glcβ1-Cer
GT1b: NeuNAcα2,3Galβ1-3GalNAcβ1-(NeuNAcα2,8NeuNAcα2,3)4Galβ1-4Glcβ1-Cer
GQ1b: NeuNAcα2-8NeuNAcα2,3Galβ1-3GalNAcβ1-(NeuNAcα2,8NeuNAcα2,3)4Galβ1-4Glcβ1-Cer
