# Supplementary material for: Neurological disorders-associated anti-glycosphingolipid IgG-antibodies display differentially restricted IgG subclass distribution
Source: Sci Rep. 2020 Aug 4;10:13074. doi: 10.1038/s41598-020-70063-5 (PMC7403582; doi:10.1038/s41598-020-70063-5)
Supplement: Supplementary file 1 — Supplementary information. [file 41598_2020_70063_MOESM1_ESM.pdf]

Supplementary Information

NEUROLOGICAL DISORDERS-ASSOCIATED ANTI-GLYCOSPHINGOLIPID IgG-ANTIBODIES DISPLAY DIFFERENTIALLY RESTRICTED IgG SUBCLASS DISTRIBUTION

R.D. Lardone [a,b,*], F.J. Irazoqui [a,b], G.A. Nores [a,b]

[a] Universidad Nacional de Córdoba. Facultad de Ciencias Químicas. Departamento de Química Biológica Ranwel Caputto. Córdoba, Argentina.

[b] CONICET. Universidad Nacional de Córdoba. Centro de Investigaciones en Química Biológica de Córdoba (CIQUIBIC), Córdoba, Argentina.

[*]**Correspondence:** Ricardo D. Lardone, Facultad de Ciencias Químicas (UNC), Ciudad Universitaria, X5000HUA Córdoba, Argentina. Phone: +54-351-5353855 (ext. 3417); E-mail: rlardone@fcq.unc.edu.ar

Supplementary Figure S1: Distribution of anti-non-self glycan and anti-self glycan IgM and IgG subclass antibodies in neurological disorder patients. HPTLC-I results from all 27 neurological disorders patients analyzed for anti-non-self glycan and anti-self glycan IgM and IgG subclasses, as described in Methods section and in Figure 1 (See Figure 1B for neurological disorder diagnosis information on each patient number. HPTLC-I for patients 1 to 5 used plates with Nt7 and "A" glycolipid antigens on left lane and the remaining antigens on right lane).

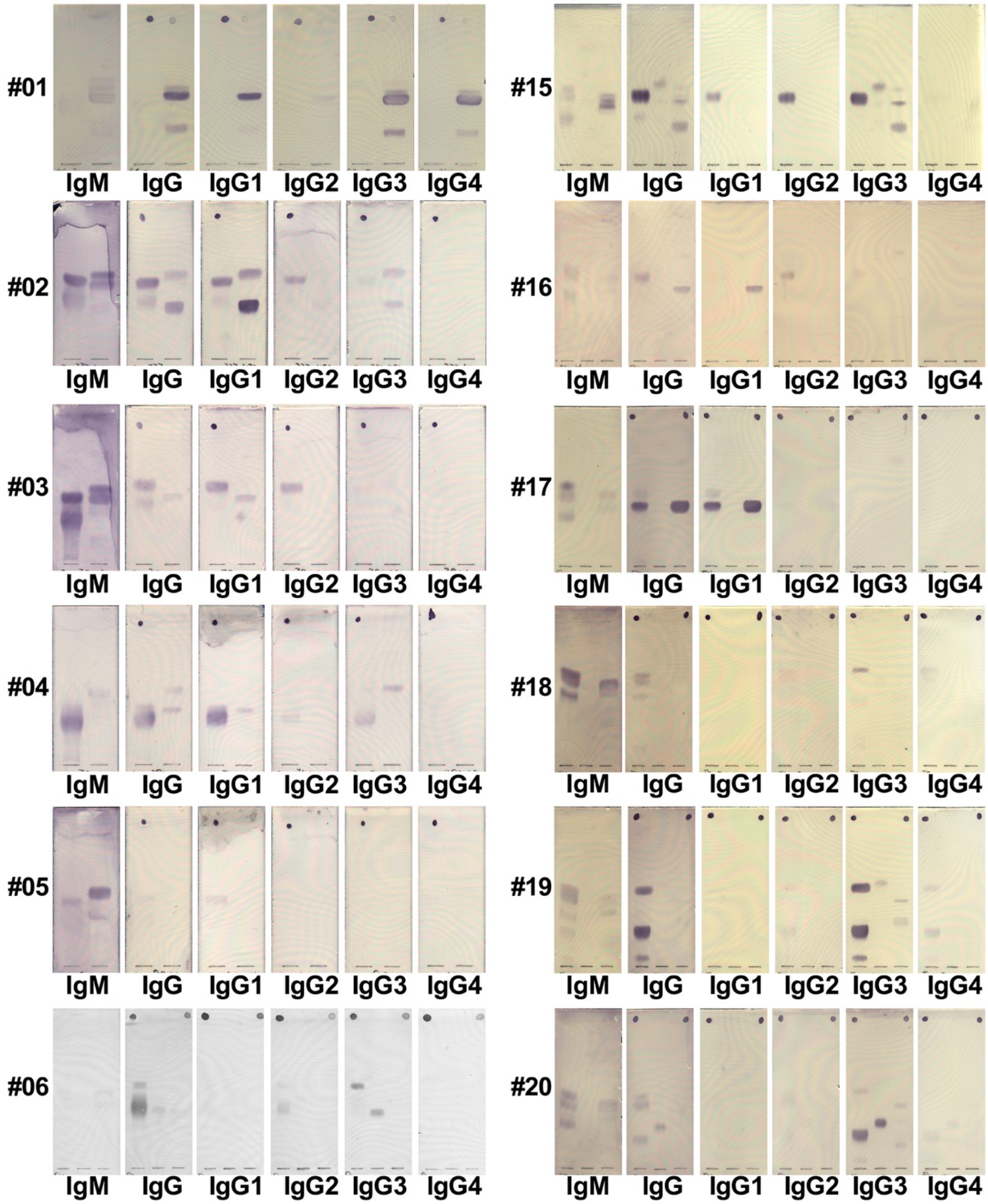

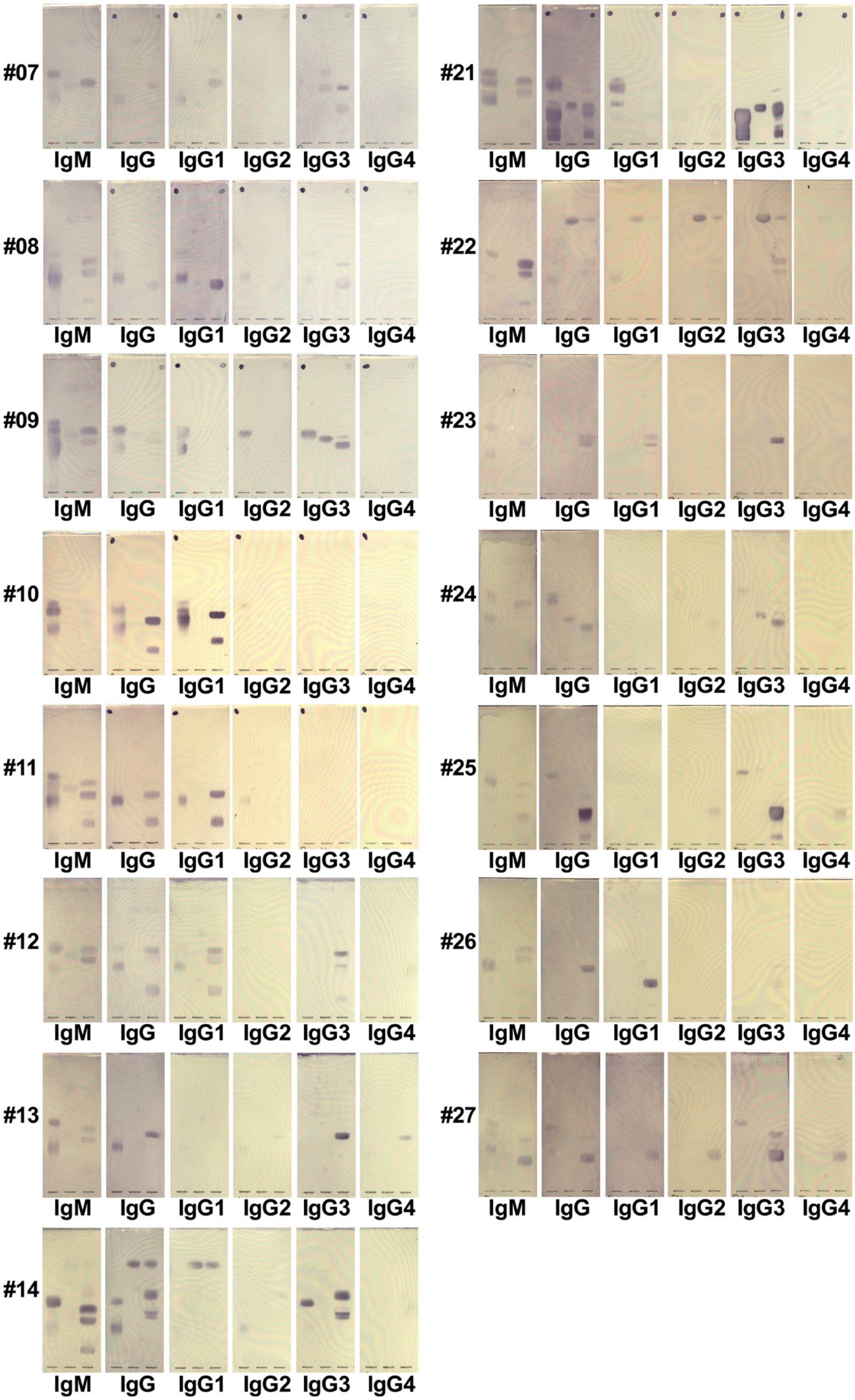
#07
IgM IgG IgG1 IgG2 IgG3 IgG4
#08
IgM IgG IgG1 IgG2 IgG3 IgG4
#09
IgM IgG IgG1 IgG2 IgG3 IgG4
#10
IgM IgG IgG1 IgG2 IgG3 IgG4
#11
IgM IgG IgG1 IgG2 IgG3 IgG4
#12
IgM IgG IgG1 IgG2 IgG3 IgG4
#13
IgM IgG IgG1 IgG2 IgG3 IgG4
#14
IgM IgG IgG1 IgG2 IgG3 IgG4
#21
IgM IgG IgG1 IgG2 IgG3 IgG4
#22
IgM IgG IgG1 IgG2 IgG3 IgG4
#23
IgM IgG IgG1 IgG2 IgG3 IgG4
#24
IgM IgG IgG1 IgG2 IgG3 IgG4
#25
IgM IgG IgG1 IgG2 IgG3 IgG4
#26
IgM IgG IgG1 IgG2 IgG3 IgG4
#27
IgM IgG IgG1 IgG2 IgG3 IgG4
